# Automated detection of type 1 ROP, type 2 ROP and A-ROP based on deep learning

**DOI:** 10.1038/s41433-024-03184-0

**Published:** 2024-06-25

**Authors:** Eşay Kıran Yenice, Caner Kara, Çağatay Berke Erdaş

**Affiliations:** 1 Department of Ophthalmology, University of Health Sciences, Etlik Zübeyde Hanım Maternity and Women’s Health Teaching and Research Hospital, Ankara, Turkey; 2 Department of Ophthalmology, Etlik City Hospital, Ankara, Turkey; 3 Department of Computer Engineering, Baskent University, Ankara, Turkey

**Keywords:** Eye diseases, Retinal diseases

## Abstract

**Purpose:**

To provide automatic detection of Type 1 retinopathy of prematurity (ROP), Type 2 ROP, and A-ROP by deep learning-based analysis of fundus images obtained by clinical examination using convolutional neural networks.

**Material and methods:**

A total of 634 fundus images of 317 premature infants born at 23–34 weeks of gestation were evaluated. After image pre-processing, we obtained a rectangular region (ROI). RegNetY002 was used for algorithm training, and stratified 10-fold cross-validation was applied during training to evaluate and standardize our model. The model’s performance was reported as accuracy and specificity and described by the receiver operating characteristic (ROC) curve and area under the curve (AUC).

**Results:**

The model achieved 0.98 accuracy and 0.98 specificity in detecting Type 2 ROP versus Type 1 ROP and A-ROP. On the other hand, as a result of the analysis of ROI regions, the model achieved 0.90 accuracy and 0.95 specificity in detecting Stage 2 ROP versus Stage 3 ROP and 0.91 accuracy and 0.92 specificity in detecting A-ROP versus Type 1 ROP. The AUC scores were 0.98 for Type 2 ROP versus Type 1 ROP and A-ROP, 0.85 for Stage 2 ROP versus Stage 3 ROP, and 0.91 for A-ROP versus Type 1 ROP.

**Conclusion:**

Our study demonstrated that ROP classification by DL-based analysis of fundus images can be distinguished with high accuracy and specificity. Integrating DL-based artificial intelligence algorithms into clinical practice may reduce the workload of ophthalmologists in the future and provide support in decision-making in the management of ROP.

## Introduction

Retinopathy of prematurity (ROP) is a vision-threatening vasoproliferative disease of the retina that occurs in premature infants with incomplete retinal vascular development [1]. The risk of ROP disease, which mostly affects newborns with low birth weight (BW) and early gestational age (GA), is increasing as a result of increasing neonatal survival rates. Therefore, the need for screening examinations increases and the importance of early diagnosis and treatment becomes more evident [2].

The clinical diagnosis has been standardized based on the International Classification of Retinopathy of Prematurity (ICROP), developed in the 1980s and revised in 2005 and 2021 [3–5]. Although there are important updates in the classification criteria in the guidelines, the disease zone, stage, and the presence of pre-plus or plus disease maintain their importance in the diagnosis of ROP. While ROP is detected early stages in most infants (stage 1 or stage 2) and regresses without any intervention, 10% may progress to Type 1 ROP and require treatment, as stated in the ETROP study [6]. On the other hand, aggressive ROP (A-ROP), which is defined by ICROP [5] as accompanied by pathological neovascularization and plus disease without progression in the typical stages of ROP, is a severe form of ROP that can result in vision loss if untreated. Therefore, accurate and timely diagnosis of ROP by clinical examination or evaluating retinal images is important.

The increasing use of fundus photographs to document ROP has increased the application of automatic image analysis in ROP, resulting in significant advances in artificial intelligence (AI) applications. Deep learning (DL), which has the potential for automatic and image-based disease diagnosis, is gaining importance as an AI application that achieves serious performances with the analysis of complex medical images. In recent years, DL algorithms using convolutional neural networks (CNNs) have guided the diagnosis of ROP by automatic analysis of fundus images without the need for a manual process. In some studies, DL algorithms have been developed for the diagnosis of ROP, such as the detection of the presence of plus disease, the ability to distinguish infants with ROP, or the vascular severity score in the diagnostic performance of ROP, based on the analysis of fundus images [7–9].

Our goal was to provide automatic detection of Type 1 ROP, Type 2 ROP, and A-ROP by DL-based analysis of fundus images obtained by clinical examination using CNNs.

## Material and methods

The present study was conducted by the principles of the Declaration of Helsinki following the approval of the Ethics Review Board (AEŞH-EK1-2023-432).

### Data sets

Three hundred seventeen premature infants who met the screening criteria according to the national screening guideline (premature infants with GA < 34 weeks and BW ≤ 1700 g or GA ≥ 34 weeks and BW > 1700 g, whose clinical condition was unstable) [10] and whose fundus images were available between January 2010-December 2022 were included in the study.

In addition to demographic information such as BW, GA, and gender, ROP stages and zones, treatment options, and postmenstrual age (PMA) at treatment were recorded. Fundus images of all premature infants taken with the Heine Video Omega® 2C indirect ophthalmoscope (Heine Optotechnik, Herrsching, Germany) were evaluated. Images were taken from the posterior pole (optic disc and macula) and peripheral retina. As stated in the ETROP study [6], laser photocoagulation (LPC) was applied to infants with Type 1 ROP and intravitreal bevacizumab (IVB) treatment was applied to infants with zone I ROP according to the results of the Bevacizumab Eliminates the Angiogenic Thread of ROP (BEAT-ROP) study [11]. Again, IVB treatment was applied to infants with posterior zone II ROP to prevent possible long-term complications that may occur after laser treatment, since the ROP line is close to the border of zone I in the posterior and has a large avascular area.

### Development of DL algorithm

CNN architectures, which consist of multiple convolutional layers, perform operations that modify data with data-specific learning properties. In the literature, there are different architectures based on the CNN principle. While there are many layers in the designs of the architectures, the related architectures differ from each other mainly in the number of layers and the size of the new data to be produced according to the parameters in the layers. In this study, RegNetY002, which is a CNN subtype, will be used [12]. The reason why this architecture is preferred is its high performance, efficiency, scalability, and innovativeness. We used the stratified 10-fold cross-validation (CV) method during the training to evaluate and standardize our model. The samples were randomly partitioned into 10 equal-sized segments with meticulous attention paid to ensuring homogeneity and similarity within each segment. In this method, each part is set aside for testing, while the remaining parts are employed for training. This iterative process continues until every part has been utilized for testing. Thus, 90% (*n* = 170 images) of the data is used for training and 10% (*n* = 19) for validation in each trial. In this way, by obtaining a homogeneous distribution in each fold and using each subset as both training and test data, it was possible to evaluate the performance of the model independently of the subsets and to reduce the risk of memorization of the model. Additionally, to compare the performance of the model under equal conditions, the model was trained using 50 epochs and 0.001 learning rate parameters in all experiments.

To develop the algorithm, a total of 634 fundus images of 317 infants were collected. In the collected images, images with poor image quality due to optical artifacts, excessive light exposure, hazy peripheral fundus images, and low-resolution images were not evaluated. Finally, a total of 189 images, 41 for A-ROP, 56 for Type 1 ROP, and 92 for Type 2 ROP, were used to detect and classify ROP. In addition, 43 and 12 images were used to detect stage 2 and stage 3 ROP from peripheral images, respectively. Fundus images are classified and labeled according to the ICROP-3 [5] diagnostic criteria, and they are also graded and labeled according to the severity of ROP: Type 1 ROP-Type 2 ROP-A-ROP and Stage 1 ROP-Stage 2 ROP and Stage 3 ROP. To increase the classification performance of these architectures, raw images were subjected to various pre-processes.

### Image pre-processing

**Fig. 1:**
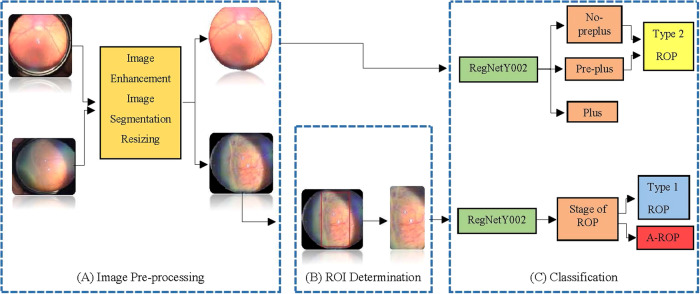
Workflow of the proposed algorithm. **A** Raw images were subjected to various pre-processing steps (image enhancement, image segmentation and resizing) in order to increase the classification performance of the model. **B** After pre-processing, the boundaries of ROP were detected in the peripheral retinal images using the Canny edge detection algorithm. Then, using the Yolo v7 detector algorithm, the detected contours were determined by red rectangles. As a result, we obtained a rectangular region (ROI). **C** ROP classification was made from the images obtained after pre-processing and ROI determination using RegNetY002.

Before analysis with DL, we processed the original images in our dataset to fit the image into the model. The pre-processing steps consisted of image enhancement, image segmentation, and resizing. First of all, the lens area in the raw images will be determined using the Adaptive Background Removal with Edge Attention algorithm, and the parts that do not contain meaningful data and have noise will be eliminated [13]. Then, Contrast Limited Adaptive Histogram Equalization (CLAHE) will be used to remove blur in the images after the segmentation process [14]. Finally, the images will be resized to 224 × 224 × 3 format to adapt to classification models (Fig. 1A). After resizing, posterior pole images were classified into two categories pre-plus and plus disease using RegNetY002 (Fig. 1C).

### Region of interest (ROI) determination

**Fig. 2:**
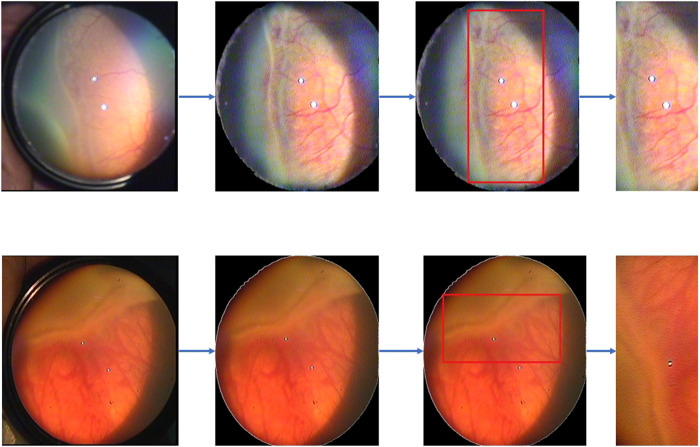
Images of the region of interest (ROI) determination process in infants with stage 2 and stage 3 ROP. The original retinal images are in the first column, the images obtained after pre-processing are in the second column, the images related to the ROP contours determined by the red rectangle (ROI) are in the third column, and the ROI area used to train the model is in the fourth column.

After pre-processing, the boundaries of ROP were detected in the peripheral retinal images using the Canny edge detection algorithm [15]. Then, using the Yolo v7 detector algorithm, the detected contours were determined by red rectangles [16]. As a result, we obtained a rectangular region (ROI) (Fig. 1B). To study the ridge line, we made sure the rectangle was on this contour. Images were then classified according to ROP staging using RegNetY002 (Fig. 1C). Images of the ROI determination process in infants with stage 2 and stage 3 ROP are shown in Fig. 2. As shown in Fig. 2, the original retinal images are in the first column, the images obtained after pre-processing are in the second column, the images related to the ROP contours determined by the red rectangle (ROI) are in the third column, and the ROI area used to train the model is in the fourth column.

As a result, after pre-processing the images, infants with stage 1 or 2 disease in zones II-III, with or without preplus disease, were classified as Type 2 ROP. Then, ROI determination was performed from peripheral retina images from the temporal side of the infants other than Type 2 ROP. In this way, infants with plus disease and ROP staging will be classified as Type 1 ROP, and infants with plus disease without ROP staging will be classified as A-ROP.

### Statistical analysis

For statistical analysis, Statistical Package for the Social Sciences (SPSS Inc., Chicago, Illinois, USA) version 25.0 was used. Categorical data were expressed as numbers (n) and percentage (%), and descriptive data as mean ± standard deviation (SD). The classification performance of the model was reported as accuracy and specificity and described graphically via the receiver operating characteristic (ROC) curve. Additionally, the quantitative performance of the model was summarized by the area under the curve (AUC).

## Results

### Clinical and demographic characteristics of infants

The study included 317 infants with a mean GA of 28 ± 2 weeks (range: 23–34 weeks) and a mean BW of 1156 ± 404 g (range: 400–2770 g). Out of 317 infants, 163 (51.4%) were female and 154 (48.6%) were male. Treatment was applied to 147 (46.4%) infants included in the study. Among the infants, anti-VEGF treatment was applied to 35 of 36 infants (97.2%) diagnosed with A-ROP, while 1 (2.8%) was treated with LPC. Again, 30 of 111 infants (27%) diagnosed with Type 1 ROP were treated with anti-VEGF therapy, while 81 (73%) were treated with LPC. Additionally, 170 (53.6%) infants were followed up with a diagnosis of Type 2 ROP, and it was observed that ROP spontaneously regressed without the need for any treatment. Infants diagnosed with A-ROP had a lower mean BW (992 ± 344, 1007 ± 286, *p* = 0.630) and an earlier PMA at treatment than infants diagnosed with Type 1 ROP (33.83 ± 1.83, 36.46 ± 2.46, *p* = 0.000).

### Performance of the DL model for ROP classification

Poor image quality due to optical artifacts, noise, excessive light exposure, etc. in fundus images may lead to incorrect evaluations. Therefore, in our study, after excluding images with low image quality, 189 images (41 for A-ROP, 56 for Type 1 ROP, and 92 for Type 2 ROP) from 317 infants were evaluated for ROP detection and classification.

The model trained using RegNetY002, a CNN classifier, was evaluated with stratified 10-fold CV on the dataset. Our model achieved 0.98 accuracy and 0.98 specificity in detecting Type 2 ROP versus Type 1 ROP and A-ROP. The model correctly detected 186 of 189 images. On the other hand, as a result of analysis of ROI regions in peripheral images, the model achieved 0.90 accuracy and 0.95 specificity in detecting stage 2 ROP versus stage 3 ROP and 0.91 accuracy and 0.92 specificity in detecting A-ROP versus Type 1 ROP. The model correctly detected 50 of 55 images for detecting Stage 2 ROP versus Stage 3 ROP and 96 of 97 images for A-ROP versus Type 1 ROP, respectively.

**Fig. 3:**
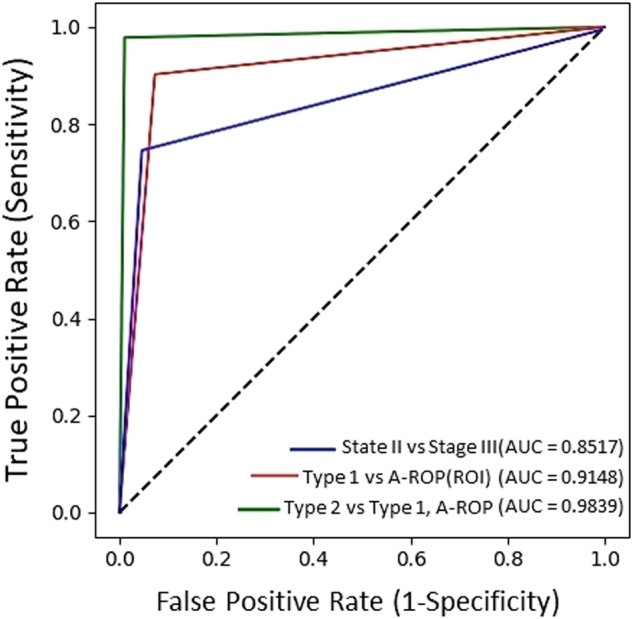
A receiver operating characteristic (ROC) curve and area under the curve (AUC) values of the trained model for ROP classification. The green line describes the detection of Type 2 ROP versus Type 1 ROP and A-ROP (AUC = 0.98). The red line describes the detection of A-ROP versus Type 1 ROP by ROI regions (AUC = 0.91). The blue line describes the detection of stage 2 ROP versus stage 3 ROP (AUC = 0.85).

The AUC scores were 0.98 for Type 2 ROP versus Type 1 ROP and A-ROP, 0.85 for Stage 2 ROP versus Stage 3 ROP, and 0.91 for A-ROP versus Type 1 ROP. The ROC curve and AUC values of the model are shown in Fig. 3.

## Discussion

This study evaluates the performance of the system in diagnosing and classifying ROP by analyzing fundus photographs based on DL algorithms using CNNs. To the best of our knowledge, although there are studies focusing on early-stage ROP or distinction of pre-plus and plus disease, there are not many studies highlighting the classification of Type 1 ROP, Type 2 ROP, and A-ROP.

Huang et al. aimed to distinguish early-stage (stage 1 ROP and stage 2 ROP) ROP from no-ROP by using a deep CNN. They showed that the proposed model can distinguish early-stage ROP with high accuracy and specificity [17]. Wang et al. developed two deep neural network (DNN) (Id-Net and Gr-Net) models for the detection of ROP from fundus images. With Id-Net, the model classified images as “Normal” and “ROP” with 96% sensitivity and 99% specificity, while with Gr-Net it graded images as “Mild” and “Severe” with 88% sensitivity and 92% specificity. However, the proposed model could not detect details such as ROP staging, the presence of plus disease, or the distinction of ROP types [7]. In the CNN model applied by Brown et al. to classify ROP as normal, pre-plus, and plus disease from retinal photographs, it was shown that plus disease could be detected with 93% sensitivity and 94% specificity, and pre-plus disease or worse could be detected with 100% sensitivity and 94% specificity. They also stated that this automated algorithm could detect plus disease in ROP with better accuracy than experts [8]. Similarly, “i-ROP” and “ROPTool” systems focus on plus disease in ROP [18–20]. 36,231 images were evaluated with the model developed by Tong et al. based on DL to automatically classify the severity of ROP from fundus images and to detect the stage of ROP and the presence of plus disease. It was stated that the trained model achieved 0.93% accuracy in classifying ROP severity (with 101-layer CNN (ResNet)), 0.95% accuracy in detecting the ROP stage (with faster region-based CNN (Faster-RCNN)), and 0.89% accuracy in detecting plus disease (with Faster-RCNN) [21]. In the automatic deep CNN-based system developed by Li et al. for the diagnosis of stage 1–3 ROP, fundus images were segmented with Retina U-Net, while images were classified (normal, stage 1, stage 2, stage 3) with Dense Net. As a result, it was stated that the developed model achieved high accuracy in detecting ROP stages. Additionally, similar to our study, the ROI area was determined in this study but was used to evaluate vascular proliferation near the demarcation line or ridge [22]. Redd et al. stated that the ROP DL (i-ROP DL) system, which they applied to detect various parameters and levels of ROP from posterior pole images and evaluate the performance, reached high accuracy in detecting clinically significant ROP (cases requiring referral; defined as Type 1 ROP, Type 2 ROP and pre-plus disease). In addition, they stated that the i-ROP DL system vascular severity score has an AUROC value of 96% in the detection of Type 1 ROP, 91% in the detection of clinically significant ROP, and 0.99% in the detection of plus disease, and as a result, the i-ROP system trained only with posterior pole images could make the correct diagnosis in identifying the disease and determining its severity [23].

In this study, we trained the system using posterior pole images and segmented images of the ROP line within the ROI. We also calculated the quantitative performance of the model. Our results suggest that the trained model achieved high accuracy, specificity, and AUC scores in ROP classification.

However, there are some limitations in the study. First, the retrospective design of the study and the small sample size. Secondly, there are no ROP patients in advanced stages such as stages 4–5 - it may have affected the distribution of the data set and therefore the performance of the model. Thirdly, the number of images available for training the model is small - excluding images with poor image quality may have affected the performance of the model and the generalizability of the results. On the other hand, the number of fundus images available affected the distribution of the infants included in the study into groups. This has caused our treatment rate to be high.

In conclusion, our study demonstrated that ROP classification by DL-based analysis of fundus images can be distinguished with high accuracy and specificity. Integrating DL-based AI algorithms into clinical practice may reduce the workload of ophthalmologists in the future and provide support in decision-making in the management of ROP.

## Summary

### What was known before

- In recent years, DL algorithms using convolutional neural networks (CNNs) have guided the diagnosis of ROP by automatic analysis of fundus images without the need for manual process.
- To the best of our knowledge, although there are studies focusing on early-stage ROP or distinction of pre-plus-plus disease in the diagnosis of ROP, there are not many studies highlighted the classification of Type 1 ROP, Type 2 ROP and A-ROP.

### What this study adds

- Our study demonstrated that ROP classification by DL-based analysis of fundus images can be distinguished with high accuracy and specificity.
- Integrating DL-based AI algorithms into clinical practice may reduce the workload of ophthalmologists in the future and provide support in decision-making in the management of ROP.

## Data Availability

The dataset used and analyzed during the current study are available from the corresponding author on reasonable request.
